# Effects of Dietary Fiber, Phenolic Compounds, and Fatty Acids on Mental Health: Possible Interactions with Genetic and Epigenetic Aspects

**DOI:** 10.3390/nu16162578

**Published:** 2024-08-06

**Authors:** Mariane Lutz, Pablo R. Moya, Sofía Gallorio, Ulises Ríos, Marcelo Arancibia

**Affiliations:** 1 Center for Translational Studies in Stress and Mental Health (C-ESTRES), Universidad de Valparaíso, Valparaíso 2360102, Chile; mariane.lutz@uv.cl (M.L.); pablo.moya@uv.cl (P.R.M.); ulises.rios@uv.cl (U.R.); 2 Department of Public Health, School of Medicine, Faculty of Medicine, Universidad de Valparaíso, Valparaíso 2362735, Chile; 3 Institute of Physiology, Faculty of Sciences, Universidad de Valparaíso, Valparaíso 2360102, Chile; 4 School of Medicine, Faculty of Medicine, Universidad de Valparaíso, Valparaíso 2362735, Chile; sofia.gallorio@alumnos.uv.cl; 5 Department of Psychiatry, School of Medicine, Faculty of Medicine, Universidad de Valparaíso, Valparaíso 2362735, Chile

**Keywords:** mental health, fiber, phenolic compounds, EPA, DHA, epigenetics, genetics

## Abstract

Scientific evidence shows that dietary patterns are a key environmental determinant of mental health. Dietary constituents can modify epigenetic patterns and thus the gene expression of relevant genetic variants in various mental health conditions. In the present work, we describe some nutrigenomic effects of dietary fiber, phenolic compounds (plant secondary metabolites), and fatty acids on mental health outcomes, with emphasis on their possible interactions with genetic and epigenetic aspects. Prebiotics, through their effects on the gut microbiota, have been associated with modulation in the neuroendocrine response to stress and the facilitation of the processing of positive emotions. Some of the genetic and epigenetic mechanisms include the serotonin neurotransmitter system (*TPH1* gene) and the brain-derived neurotrophic factor (inhibition of histone deacetylases). The consumption of phenolic compounds exerts a positive role in neurocognitive domains. The evidence showing the involvement of genetic and epigenetic factors comes mainly from animal models, highlighting the role of epigenetic mechanisms through miRNAs and methyltransferases as well as the effect on the expression of apoptotic-related genes. Long-chain *n*-3 fatty acids (EPA and DHA) have been mainly related to psychotic and mood disorders, but the genetic and epigenetic evidence is scarce. Studies on the genetic and epigenetic basis of these interactions need to be promoted to move towards a precision and personalized approach to medicine.

## 1. Introduction

Mental health is an intrinsic part of individual and collective health and well-being, and is considered a basic human right [1]. Epidemiological studies show that mental health disorders have a high prevalence [2] and burden of disease [3]. Because health interventions in the area are insufficient, the focus of attention should be directed towards prevention and early detection. One approach for research and eventual intervention is the relationship between diet and mental health. The functioning of the central nervous system requires an adequate supply of nutrients and bioactive compounds that contribute to its structural integrity and optimal function [4].

This article describes some of the relationships between dietary patterns and mental disorders, based on evidence provided by preclinical and human studies in the field of neuro-nutrigenomics. Given the multiplicity of food components that affect proper brain function, some aspects related to phenolic compounds are emphasized due to their antioxidant and anti-inflammatory actions. For example, the polyunsaturated fatty acids EPA (eicosapentaenoic acid, C20:5*n*-3) and DHA (docosahexaenoic acid, C22:6*n*-3), which are part of the nervous tissue membrane structure that modulate its functioning and give rise to neuroprotective metabolites; and prebiotic fiber, which determines intestinal eubiosis (i.e., the adequate state of the intestinal microbiota) and the proper functioning of the gut–brain axis. Initially, some general characteristics of these dietary components are described, as well as the available evidence regarding their role in mental health, emphasizing their interaction at the genetic and epigenetic levels.

## 2. Dietary Patterns and Mental Health

Nutritional psychiatry is an interdisciplinary area of growing interest [5]. Mental health status determines lifestyles, including dietary patterns, which, in turn, affect mental health throughout the life cycle [6], generating a complex bidirectional association. Food and its constituents not only affect brain structures and functions but also the profile of gastrointestinal hormones and peptides, neuropeptides, neurotransmitters, and other cellular signals, as well as the gut microbiota, a set of elements that, in a coordinated way, affect mental health [7,8]. Food quality is part of our lifestyle. Among the many actions performed by food components, their ability to affect gene expression stands out. Although genetics plays a major role in the predisposition to develop certain mental health disorders, the role of epigenetic determinants is increasingly recognized, including the type of diet, which can be classified as healthy or not depending on the effects it causes. In fact, nutrients and bioactive compounds may exert epigenetic modifications, affecting a variety of diseases [9,10]. The dietary pattern, shaped by the most frequently consumed foods, is considered healthy if it satisfies physiological needs and exerts beneficial effects on the body [11,12]. A healthy and sustainable dietary pattern reduces risk factors for noncommunicable diseases, increases life expectancy, reduces associated morbidity and mortality, and is associated with a lower public health cost [13].

The main constituents that contribute to reducing the risk of noncommunicable diseases are found in plant-based foods. Unhealthy diets, on the other hand, are associated with high energy intake, red and processed meats, refined sugars and flours, sodium, and saturated and trans fatty acids [12,14]. Globalized diets are characterized by high energy density and low nutrient density, a high consumption of ultra-processed foods, and malnutrition due to the excess of calories coexisting with undernutrition. 

Dietary patterns recognized as healthy have scientific evidence of their effects [15], as is the case of the Mediterranean diet, DASH (Dietary Approaches to Stop Hypertension), and MIND (Mediterranean-DASH Intervention for Neurodegenerative Delay) diets, the latter as a mixture of the first two [16,17,18]. These diets are low in digestible carbohydrates (mainly starches) and saturated fats and discourage the consumption of ultra-processed, high-sugar, sodium, or low-fiber foods. In general, they contribute to the reduction of adverse oxidative, inflammatory, and metabolic processes, improve endothelial function, reduce the production of pro-inflammatory cytokines, and reduce the triggers of metabolic syndrome [19]. Evidence associates these diets with a lower incidence of mental illness, including depressive disorders (DD) [19,20] and neurocognitive decline [21], which has led to the recognition of their clinical importance [22].

Healthy eating patterns contain various nutrients and bioactive compounds that interact, generating synergies. Evidence about the effects of consuming these components comes mainly from in vitro and in vivo trials and observational studies, including prospective ones [13,23,24]. Clinical trials involving dietary intervention, meanwhile, are still scarce, although they are increasingly being developed.

## 3. General Characteristics of Food Components in Mental Health

### 3.1. Fiber

Fiber is mainly made up of polysaccharides from the plant cell wall. As these compounds are not digestible, many of them are fermented by the gut microbiota [25] (GM). Its consumption is essential for the maintenance of eubiosis [26]. Microbial fermentation mainly generates short-chain fatty acids (SCFA) such as acetic, propionic, and butyric acids and branched-chain amino acids [27], which promote eubiosis and stimulate the release of neuropeptides, hormones, and other molecules from enteroendocrine cells [28]. Additionally, the GM generates lipopolysaccharides, bile acids, and catecholamines and induces the secretion of neurotransmitters [29]. 

Together with the brain, the gut constitutes a dynamic and complex bidirectional axis of action, the alteration of which would be associated with mental health disorders [30,31,32] and affect eating behavior [33,34,35,36]. Likewise, dysbiosis, or abnormal alteration of the composition and abundance of GM [37], is associated with oxy-inflammation, cell degeneration, and changes in the blood–brain barrier, favoring neuro-deterioration [38,39]. The widely described association between GM, DD, and anxiety disorders [40,41] is attributed to various mechanisms, including the generation of metabolites other than those coming from adequate GM, loss of intestinal barrier integrity, reduced mucus secretion, and translocation of pathogenic microorganisms and toxins into the bloodstream, with local and systemic inflammatory responses [42]. In a cohort study with biobank data, Amin et al. [43] evaluated the metabolomic profile of GM and its association with major DD, identifying 124 metabolites of energy metabolism and lipid pathways whose levels are altered, which are associated with changes in the composition of GM in affected patients, which are associated with dysbiosis. GM modulates signaling pathways, such as glucagon-like peptide (GLP-1) or peptide YY, and activates reward signals [44]. The metabolites generated (“psychobiotics”) can produce epigenetic changes by DNA methylation (i.e., repression of gene expression of the methylated region), covalent histone modifications (i.e., repression or promotion of gene expression according to the type of histone modification), and by miRNAs (i.e., by diminishing the translation efficiency of mRNAs or promoting their degradation) [45]. For example, in a mouse model, butyrate generated in the colon is a modulator of mood and behavior [46] by inhibiting histone acetylation. GM also generates neuroactive metabolites, such as serotonin and gamma-aminobutyric acid (GABA), that affect central appetite control [47] and can stimulate other systems, such as the endocannabinoid [48]. In a trial with dietary supplementation with prebiotic fiber in the form of fructo-oligosaccharides (FOS) or galacto-oligosaccharides (GOS), Schmidt et al. [49] observed that the consumption of GOS produced a lower neuroendocrine response to stress and greater processing of positive responses to emotional stimuli, similar to that obtained with citalopram or diazepam, in healthy people. The results are close to the evidence obtained in animal models, where these prebiotics showed anxiolytic and antidepressant effects, reversing the impact of chronic stress [50].

### 3.2. Phenolic Compounds (PC) or Polyphenols

**Figure 1:**
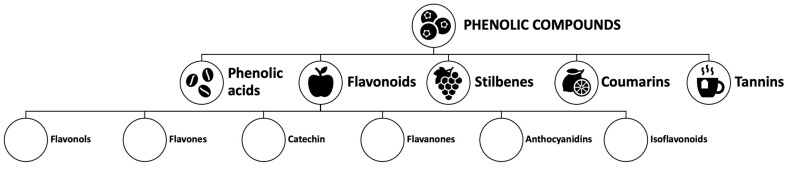
General structures of phenolic compounds in plants.

PC correspond to secondary metabolites of plants, which exert multiple actions in the body. Among the PC whose effects on mental health have been described are flavonoids, phenolic acids, stilbenes, and complex molecules, such as tannins and lignans. The most abundant PC in foods are flavonoids, antioxidants whose presence varies in quality and quantity, highlighting red fruits, tea, coffee, cocoa, citrus fruits, and soy, among others, as good sources. In general, the bioavailability of polyphenols is low [51,52], and to act in the brain, they must pass through the blood–brain barrier [53]. Their high structural diversity complicates their intestinal bioaccessibility (release from the food matrix) and their absorption, which is very low, so most of them go directly to the colon, where they are metabolized by the microbiome [54]. Figure 1 depicts the main families of PC.

Due to their antioxidant and anti-inflammatory actions, PC contribute to reducing the risk of cognitive decline. Effects such as improvement in memory, language, attention, concentration, psychomotor speed, and other cognitive domains have been demonstrated, in addition to increasing blood perfusion in brain areas associated with Alzheimer’s disease (AD) [55]. Additionally, they are neuroprotective and stimulate neuroplasticity by inhibiting oxy-inflammation and activating synaptogenesis, neurogenesis pathways [56,57] and antiplatelet therapy (i.e., the therapy used to reduce the activation of platelets, which is a risk factor for various diseases) [58], actions that are related to neuroprotection [59]. They act as direct antioxidants (trapping free radicals) and indirect antioxidants (stimulating the synthesis of endogenous antioxidant enzymes), helping to reduce the risk of cognitive decline associated with oxidative stress, in addition to multiple other actions, which is why they are considered pleiotropic [60]. PC induce nuclear factor Nrf2 and inhibit nuclear factor kappa B (NF-κB), a transcription factor responsible for the expression of immune response genes, reducing the expression of pro-inflammatory mediators, among other actions [61,62]. Overactivation of NF-κB induces neurotoxicity and promotes neuronal death by increasing pro-inflammatory cytokines, such as IL-6, TNF-α, and cyclooxygenase-2 (COX2) activity [63].

Neuropathological changes leading to AD include the deposition of amyloid precursor protein and misfolded β-amyloid (Aβ)-protein-derived peptides or amyloid plaques in the brain parenchyma and the appearance of neurofibrillary aggregates of hyperphosphorylated tau protein [64]. The assembly of Aβ oligomers alters synaptic function and brain plasticity [65]. Anthocyanins, proanthocyanidins, flavan-3-ols, and catechins protect against these alterations [66]. These polyphenols are abundant in berries and are involved in reducing the development of various psychiatric pathologies, including anxiety–DD and dementias [67,68].

It should be noted that people consume a variety of foods and not their components in isolation, so the effects of a diet involve multiple interactions between nutrients and the bioactive compounds in all of them. As an example, PC significantly affect GM in a bidirectional way as microorganisms metabolize ingested polyphenols [69], affecting the functioning of the gut–brain axis. The consumption of different foods allows synergies between their components [70], so the overall effect of the dietary pattern is more important than considering some food components, which constitutes a reductionist approach to their action on mental health. Moreover, PC modulate gene expression through the regulation of specific epigenetic mechanisms, affecting metabolic processes involved in mental diseases [71].

### 3.3. EPA and DHA 

EPA and DHA are the main polyunsaturated fatty acids (PUFA) that exert important actions in the brain. Both are pre-formed in fish and shellfish, so the pescatarian diet is considered healthy. Although they can be synthesized from *a*-linolenic acid (C18:3*n*-3) of terrestrial origin (seeds, nuts, green leafy vegetables), bioconversion to EPA and DHA is very inefficient in the human body [72]. These long-chain fatty acids play structural roles in the phospholipids of brain membranes, determining their fluidity, structure, and functioning and exert various favorable metabolic effects on triglyceride levels, blood pressure, arrhythmias, inflammation, platelet aggregation, endothelial dysfunction, and modulation of the immune response, among others, in addition to their direct actions at the brain level [73,74].

DHA is the most abundant fatty acid in brain membranes, playing major roles in synaptogenesis, neurotransmission, and, therefore, neurocognition [75,76]. It participates in the expression of multiple genes [77], acting on the family of retinoid X receptors, retinoic acid receptors, and peroxisome proliferator activators [78]. Its deficiency is associated with an increased risk of mental disorders such as attention deficit disorder, dyslexia, DD, bipolar disorder, and schizophrenia [79,80]. Its mechanisms of action involve the modulation of the action of noradrenaline, dopamine, and serotonin (i.e., reuptake, synthesis, degradation, and receptor binding), anti-inflammatory, anti-apoptotic, and neurogenesis effects by regulating the synthesis of brain-derived neurotrophic factor (BDNF) [22,81].

Given the relevance of inflammatory processes in DD [82,83], one of the most outstanding preventative actions of EPA and DHA is anti-inflammation by inhibiting the release of cytokines (interferon-γ, TNF-α, IL-1β, IL-2, IL-6) acting directly on the transcription factor NF-κβ [84]. EPA and DHA generate resolvins of the E (RvE) and D (RvD) series, respectively, which disrupt the inflammatory cascade [85], and maresins (such as MaR1), which promote tissue regeneration [86]. Among the neuroprotective metabolites of DHA, neuroprotectin D1 (NPD1) also stands out, which modulates neuroinflammation and neuroprotection in aging brain cells [87].

A summary of the dietary components described, and examples of their food sources are presented in Table 1.

## 4. Genetic and Epigenetic Aspects

Currently, there is increasing evidence of the interaction between different food components and gene expression, mediated by epigenetic phenomena. Although human studies are scarce, they suggest some mechanisms with biological plausibility.

Fiber strengthens the intestinal barrier by promoting microbial proliferation and eubiosis. An example of this action is the synthesis of indole, which induces the expression of genes associated with barrier resistance through tight junctions [42]. Conversely, increased permeability allows the transfer of lipopolysaccharides and endotoxins into the circulation, promoting neurodegeneration by activating the inflammatory response [88]. This process could be mediated by an increase in the activity of the hypothalamic-pituitary-adrenal axis, which has been linked to the onset of depressive symptoms [89]. Some types of bacteria synthesize molecules that regulate serotonin synthesis by inducing *TPH1* gene expression in chromaffin cells of the gastrointestinal tract in murine models [90]. *TPH1* gene encodes the enzyme tryptophan hydroxylase isoform 1, which is involved in peripheral serotonin synthesis [90]. Some genetic variants of *TPH1* have been implicated in DD and suicidality. Indeed, genotypes AA on intron 7 and AA on the *TPH1* promoter region were shown to be predictors of suicide attempts at one year in a cohort of 343 people with mood disorders [91]. The SCFA Valeric acid has been reported to promote *TPH1* expression in enteric serotonergic neurons; however, it is not known whether this effect occurs in mesencephalic serotonergic neurons, whose serotonin levels are primarily regulated by *TPH2* (tryptophan hydroxylase isoform 2). On the other hand, at the epigenetic level, SCFA generated by intestinal fermentation are inhibitors of histone deacetylases, whose activity modifies gene expression [92]. In a mouse model, SCFA were associated with histone hyperacetylation, increasing the expression of BDNF [93], which plays a central role in the pathophysiology of DD [93]. Despite these observations, the evidence in humans is still incipient. A randomized clinical trial that analyzed the effects of the consumption of the prebiotics FOS and GOS on markers of stress and inflammation and depressive symptomatology did not verify differences between these variables when compared to the control group, but a change in the composition of the GM was exhibited, with an increase in the presence of *Bifidobacterium* [94]. Therefore, in humans, the consumption of prebiotics has an impact on the composition of GM, but their effect on clinical parameters is being studied.

PC affect various cell signaling pathways by modulating gene expression, affecting the activity of transcription factors, and modulating the synthesis of miRNAs, an effect that has been observed mainly in in vitro and animal models of cancer [95]. Research in humans on the interaction between polyphenol consumption, epigenetic modifications, and mental health outcomes is very limited. However, evidence in animal models and in vitro has yielded some potential mechanisms. Tea and coffee, two of the most consumed beverages in the world, contain various PC, which have been attributed to the modulation of gene expression via epigenetic mechanisms. Some in vitro studies have shown that the polyphenols extracted from both products exert inhibitory activity on Dnmt3a methyltransferase, which catalyzes the DNA methylation reaction, one of the mechanisms most involved in the epigenetics of neuropsychiatric disorders [96]. In a mouse model of DD, Wang et al. [97] verified that resveratrol, abundant in black grapes, reduced depressive behaviors, a phenomenon that would possibly be mediated by the inhibition of apoptosis of hippocampal neurons through the transduction pathway mediated by netrin 1 (a protein involved in apoptosis) and regulated by cyclic adenosine monophosphate (cAMP). The genetic mechanism behind the inhibition of apoptosis involved the upregulation of some apoptotic-related genes such as *P53*, B-cell lymphoma-2 (*Bcl-2*), and *Bcl-2*-associated death promoter, and the downregulation of the cleaved caspase-3 and caspase-9.

The association between suboptimal levels of PUFA and the presence of psychotic disorders has been replicated in epidemiological and meta-analytic studies [98]. In fact, it has been proposed that the lipid composition of the erythrocyte membranes of people at high risk of developing psychosis is a predictive biomarker [99], while EPA and DHA supplementation is a potential intervention for this group [100]. Jones et al. [101] conducted a Mendelian randomization study based on genomic scanning that analyzed the relationship between schizophrenia and PUFA. The authors concluded that both *n*-3 and *n*-6 long-chain PUFAs would have a protective role in schizophrenia, suggesting a potential alteration in the process of conversion of SCFA to PUFA in this population. However, from the cohort of The Avon Longitudinal Study of Parents and Children (ALSPAC), Thompson et al. [102] did not observe a significant effect of genetic variants interacting with the levels of these PUFA on the risk of psychotic symptoms in a cohort of children and adolescents. Using data from the same cohort, Sallis et al. [103], using a Mendelian randomization model, reached similar conclusions regarding perinatal depression. Along these lines, there is secondary evidence showing an inverse correlation between levels of PUFA and the presence of DD [104,105]. This relationship could be mediated by genetic factors [106], due to the existence of genetic variants of phospholipase A2 (PLA2) that determine an alteration in the metabolism of *n*-3 PUFA, which could predispose to depressive symptoms. Since this association comes from cross-sectional studies, in a different sense, low levels of *n*-3 PUFA in subjects with DD could be the result of low dietary intake. Indeed, by focusing the analysis on the genetic contribution to the relationship between EPA and DHA levels and depression, Milaneschi et al. [107] did not find consistent results from the analysis of data provided by genomic scanning studies and the construction of polygenic risk scores. Regarding neurocognitive disorders, a pilot study with EPA and DHA supplementation showed variable results on inflammatory gene expression in patients with mild neurocognitive disorder and these *n*-3 PUFA supplementation [108]. Table 2 summarizes some food constituents whose insufficiency is reported to exert genetic and epigenetic effects affecting mental health.

## 5. Future Perspectives and Limitations

The current scientific evidence supports the association between dietary eating patterns and multiple mental health outcomes. However, most of the evidence is preclinical in nature, while human studies are predominantly observational, which represents limitations for their practical application. There are only a few results from dietary intervention designs, although these are increasing. It is expected that, in the near future, studies on the genetic and epigenetic basis of these interactions will be promoted. This knowledge would allow us to move towards a precise and personalized approach to medicine.

## 6. Conclusions

International dietary intake recommendations point to healthy dietary patterns, which correspond to plant-based and pescetarian diets, the main contributors to the protective food components described. It should be borne in mind that the diet is chemically very complex, as it shows great molecular diversity, which makes it difficult to interpret the results of the studies and their clinical translation. 

The diet is made up of multiple foods whose constituents interact with each other in multiple ways, which makes research in this area difficult. Despite this, numerous dietary components have been identified as determining factors in proper brain function and mental health. Prebiotic fiber, through its effects on GM, has been associated with a modulation in the neuroendocrine response to stress and the facilitation of the processing of positive emotions. Some genetic and epigenetic mechanisms involved are the regulation of the expression of genes linked to serotonin metabolism and the synthesis of BDNF. On the other hand, the consumption of PC exerts a positive role in different neurocognitive domains. However, the evidence showing the involvement of genetic and epigenetic factors comes from animal models. Finally, EPA and DHA have been mainly related to psychotic and mood disorders, but the genetic and epigenetic basis of this association is still not well understood.

## Figures and Tables

**Table 1:** Food components that may exert beneficial effects on mental health through genetic/epigenetic mechanisms.

Food Component	Molecules	Food Sources	
Fermentable fiber	Oligosaccharides (FOS, fructooligosaccharides; GOS, galactooligosaccharides)	Legumes, onion, garlic, asparagus, chicory root, and kefir	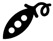
Phenolic compounds	Flavonoids (catechins, anthocyanins, and flavones)	Berries, tea, coffee, cocoa, soy, and extra-virgin olive oil	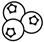
Long chain n-3 PUFA	EPA (eicosapentaenoic acid); DHA (docosahexaenoic acid)	Fish, shellfish, and algae	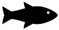

**Table 2:** Food constituents whose insufficiency is reported to exert genetic and epigenetic effects affecting mental health.

Deficient Intake	Food Sources	Specific Components	Epigenetic Mechanism	Effect on Mental Health	References
Fermentable fiber (Oligosaccharides)	Whole grains legumes, F and V, nuts, and plant-based diets	SCFA, psychobiotics (butyrate)	- GM alteration (dysbiosis) - (-) histone methylation - Some bacteria synthesize molecules that induce *TPH1* expression in chromaffin cells, which encodes tryptophan hydroxylase isoform 1, involved in serotonin synthesis. *TPH1* gene variants have been implicated in DD and suicidality. - SCFA generated by fermentation inhibit histone deacetylases, whose activity modifies gene expression, and are associated with histone hyperacetylation, increasing the expression of BDNF, affecting the pathophysiology of DD.	- DD and anxiety disorders	[40,41,42,43,44,45,46,90,91,92,93]
Phenolic compounds	F and V, legumes, extra-virgin olive oil, berries, grapes, tea, coffee, cocoa, nuts, Mediterranean diet, DASH, and MIND	Flavonoids, phenolic acids, stilbenes, tannins, and lignans	- Modulation of gene expression through the regulation of specific epigenetic mechanisms, affecting metabolic processes involved in mental diseases. - In vitro studies have shown that PC extracted from tea and coffee inhibit Dnmt3a, which catalyzes DNA methylation, mechanisms involved in the epigenetics of neuropsychiatric disorders. - Mouse model of DD: resveratrol, abundant in black grapes, reduced depressive behaviors, possibly mediated by the inhibition of apoptosis of hippocampal neurons, through the transduction pathway mediated by netrin 1. The genetic mechanism would involve the upregulation of *P53*, *Bcl-2*, and *Bcl-2*-associated death promoter, and the downregulation of the cleaved caspase-3 and caspase-9. - Modulation of GM, affecting the functioning of the gut–brain axis.	- Neuroprotection, reduction of cognitive decline - Prevention of neuropathological changes leading to AD and other dementias - Reduction of risk factors of anxiety–DD.	[55,56,57,58,59,60,61,62,63,64,65,66,67,68,69,96,97]
Long-chain *n*-3 PUFA	Fish, shellfish, seafood, algae, Mediterranean diet, DASH, and MIND	EPA (C20:5*n*-3) and DHA (C22:6*n*-3)	- *n*-3 PUFA would have a protective role in schizophrenia, in which there would be an alteration in the conversion of SCFA to PUFA. - In the ALSPAC cohort no significant effect of genetic variants interacting with the levels of these PUFA were observed on the risk of psychotic symptoms or perinatal depression. - The inverse correlation between levels of PUFA and the presence of DD could be mediated by genetic variants of PLA2, that determine an alteration in the metabolism of n-3 PUFA, which could predispose to depressive symptoms.	- DHA deficiency is associated with an increased risk of attention deficit, dyslexia, DD, bipolar disorder, and schizophrenia.	[101,102,103,104,105,106]

## Abbreviations

ALSPAC, Avon Longitudinal Study of Parents and Children; BDNF, brain-derived neurotrophic factor; cAMP, cyclic adenosine monophosphate; COX2, cyclooxygenase 2; DASH, Dietary Approaches to Stop Hypertension; DD, depressive disorders; DHA, docosahexaenoic acid; DNA, deoxyribonucleic acid; EPA, eicosapentaenoic acid; FOS, fructo-oligosaccharides; GABA, gamma-aminobutyric acid; GLP-1, glucagon-like peptide; GM, gut microbiota; GOS, galacto-oligosaccharides; MIND, Mediterranean-DASH Intervention for Neurodegenerative Delay; mRNAs, messenger ribonucleic acids; miRNAs, micro ribonucleic acids; SCFA, short-chain fatty acids; PC, phenolic compounds; NF-κB, nuclear factor kappa B; NPD1, neuroprotectin D1; PUFA, polyunsaturated fatty acids; Tph2, tryptophan hydroxylase.
